# Effects of Alcohol Consumption on Various Systems of the Human Body: A Systematic Review

**DOI:** 10.7759/cureus.30057

**Published:** 2022-10-08

**Authors:** Jerin Varghese, Sarika Dakhode

**Affiliations:** 1 Medical School, Jawaharlal Nehru Medical College, Datta Meghe Institute of Medical Sciences, Wardha, IND; 2 Community Medicine, Jawaharlal Nehru Medical College, Datta Meghe Institute of Medical Sciences, Wardha, IND

**Keywords:** various systems of human body, harmful effects, alcohol abuse, effects on general health, alcohol misuse

## Abstract

Prolonged alcohol intake for many years has been known to cause serious ailments in human beings since time memorial. Even after knowing that this dangerous addiction paves the way to one’s own grave, there isn’t much difference in the way the community sees this deadly habit. Time and again history has proven that this fatal addiction could make the life of those who consume it terrible. Also, the lives of the dear ones of alcoholic people are affected as alcohol not only affects those who consume them but also kin and friends. Various research studies conducted over many years clearly show the association of prolonged alcohol intake in the causation, aggravation, worsening, and deterioration of the health of its consumers. Moreover, chronic alcohol intake single-handedly is one of the major etiological factors in various serious diseases.

## Introduction and background

Through the ages, alcoholism has been undisputedly maintaining its position in the list of risk factors for preventable diseases in the world. According to a WHO report, 5.3% of all deaths that occurred worldwide in the year 2016 were because of harmful alcohol use [1]. It is the main culprit behind the advancing nature of many chronic diseases. It drastically increases the severity of diseases and also makes the treatments less effective. Alcohol not only affects the person physiologically, but it has many adverse effects psychologically and socially too. Also, the habit of alcoholism leads to huge expenses [2]. Apart from systemic involvement, which causes various clinical manifestations, there are certain signs and symptoms that are most of the times non-specific and that as such don’t point out or say lead to a particular diagnosis, such as nausea, agitation, vomiting, anxiety, diaphoresis, tremors, headache, visual hallucinations, tachycardia, seizures, delirium, temperature elevation, etc. It is not always necessary that these mentioned signs and symptoms are compulsorily linked with disease conditions.

Alcohol clearly plays a very important role in making many other diseases progress to their advanced stages. It has been also noted that alcohol intake and its related disorders are often associated with many other manifestations; for example, patients with alcoholic neuropathy often have associated nutritional deficiencies. Recent studies have clearly proved that alcoholism is associated with many types of cancers too and this understanding of alcoholism has spurred research minds all over the globe to find out the exact pathophysiology behind the same. Alcohol is a very easily available source of addiction, which is one of the main reasons why it remains a serious threat to the community. There is a huge variety that is available as far as alcoholic drinks are concerned. Alcohol is also one of the cheaply accessible means of addiction; this explains why alcoholism is so prevalent. A person may initially start consuming alcohol in very low amounts most probably with just a desire to try it, but once he or she gets addicted, then getting rid of the habit becomes extremely difficult. Even if a person is mentally resolute enough to quit alcoholism, his or her body, which has been modified because of the chronic use of alcohol, won’t be up to the challenge anytime soon; he or she has to overcome many hurdles put forward by the body, which could in an umbrella term be referred to as alcohol withdrawal syndrome.

There are many social stigmas associated with alcohol intake. Most people get into this addiction by getting inspired by the people whom they admire, like actors, celebrities, role models, etc. Also, exposure to the sight of family members, relatives and friends drinking alcohol has a huge impact on one’s mindset as he or she may take it to be something that is normal. In the long run, most of the time, even without their realization, people get pathetically trapped in this dangerous fatal habit of alcoholism, which eventually makes their lives pitiful in almost all aspects. Studies have shown that alcohol is also a key player in many other domains too like accidents, suicide, depression, hallucinations, violence, memory disturbances, etc.

**Figure 1:**
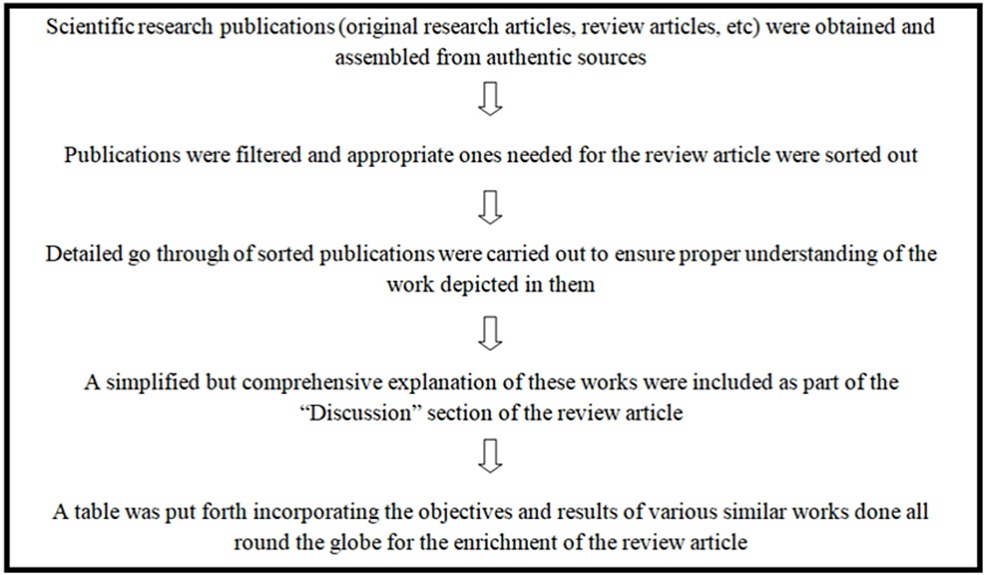
A flowchart of the methodology used for the review

The main purpose of this review article is to enable any person reading this article to get a comprehensive insight into the effects of alcohol on the various systems of the human body, and for the same, many recognized research articles published in numerous well-acknowledged journals across the globe are reviewed. The article is written using very basic and simple terminologies so that even a layperson who reads it would be able to understand it. For the easy acceptability and understanding of the reader, the discussion is written in such a way that almost every major system is reviewed one by one and the effect of alcohol on these systems put forward in very simple language. The strategies used for the establishment of this review article are summarised in Figure 1; these include considering research articles that have been published in journals with are indexed in reputed platforms, segregating articles according to the different systems, framing the review like a discussion section of an article where details are explained in simple and straight forward sentences, etc.

## Review

Impact of alcohol on the central nervous system (CNS)

Alcohol exerts various effects on our CNS in various ways, the common ones being depression of the CNS, destruction of the brain cells, contraction of the tissues of the brain, suppression of the excitatory nerve pathway activity, neuronal injury, etc [3]. Alcohol’s impact on the functioning of the brain ranges from mild and anxiolytic disinhibitory effects, motor incoordination, sedation, emesis, amnesia, hypnosis and ultimately unconsciousness [4]. The synaptic transmission is heavily disturbed and altered by ethanol, and the intrinsic excitability in various areas of the brain is also compromised. The effects of ethanol may be pre-synaptic, post-synaptic, and at times, non-synaptic too. Alcohol being a psychotropic depressant of the CNS exerts a deeply profound impact on the neurons, which alters the biological and behavioural well-being of the one who consumes it by the promotion of interference in various neuronal pathways [5]. The treatments of many disorders of the CNS are shown to be affected by the consumption of alcohol, and thus, it is generally advised to keep oneself away from alcohol if one is undergoing treatment for any CNS manifestations, like anxiety or mood disorders [6].

Alcohol use disorder (AUD) is chronic in nature and is characterized by uncontrolled drinking and also a preoccupation with alcohol. The severity of AUD is a crucial factor in how it is going to affect the human body. AUD can be mild, moderate, or severe according to the symptoms a person experiences. The clinical manifestations of AUD include signs and symptoms such as inability to control the amount of alcohol intake, spending a lot of time drinking, feeling an uncontrollable craving for alcohol, loss of interest in social activities, failure to fulfil tasks within the time provided, etc. Most of the time, along with the person who consumes alcohol, several other factors are also to be taken care of in order to effectively manage alcohol-related health conditions. These factors can be social, environmental, genetic, psychological, etc, which make a considerable impact on how alcohol affects the behaviour and body of those consuming it. Binge drinking, i.e., drinking to such an extent on a single occasion that the blood alcohol concentration level becomes 0.08% or more, is a very relevant aspect of alcohol intake, which has to be dealt with, with utmost urgency. Certain research studies suggest that mild to moderate alcohol intake provides a certain sort of protection against a few CNS disorders like dementia, ischemia of neurons, etc, but this in no way should encourage the community in promoting alcohol intake as in reality, it is very difficult to remain within the limits of mild to moderate alcohol intake, and thus, eventually, people do end up as full-time severe alcohol abusers. Epilepsy, a seizure disorder caused by disturbed nerve cell activity in the brain, aggravates on excessive alcohol intake as alcohol increases the frequency of seizures in patients of epilepsy [7]. The issue becomes more severe in those epileptic patients who have refractory forms of epilepsy. As far as comorbidities are concerned, a valid history of abuse of substances or alcohol dependence is believed to be strongly associated with a high risk of sudden unexpected death in epilepsy (SUDEP) [8]. Heavy alcohol drinking over a long period of time has been found to have an intensely negative undesirable effect on the autonomic nervous system too.

Impact of alcohol on the cardiovascular system (CVS)

Chronic alcohol intake is undoubtedly a very important risk factor as far as cardiovascular diseases are concerned and several clinical trials do point out this fact. The results of several research studies conducted in various settings clearly indicate that increased intake of alcohol has increased adverse effects on our heart and its vasculature. Alcohol exerts its action on the cardiovascular system both directly and indirectly. Blood pressure, a very vital player in the domain of cardiovascular diseases, is in turn itself affected by increased alcohol consumption. Blood pressure gets increased on regular consumption of alcohol in a manner which is dose-dependent, which in turn increases the risk of hypertension and eventually leads to various cardiovascular complications. How exactly alcohol causes hypertension is still unclear with many pathophysiological theories out there. Atrial fibrillation, one of the most common causes of arrhythmia, is associated with the high-volume chronic intake of alcohol and above 14 g alcohol/day, the relative risk dramatically increases by 10% for each extra standard drink (14 g ethanol) [9].

Cerebrovascular accidents are increased to a great extent at almost all levels of alcohol intake [10]. Alcohol intake leads to both acute (depresses the cardiac function and also alters the blood flow of the involved region) and chronic cardiovascular manifestations [11]. Alcohol abuse along with other associated factors is one of the leading causes of secondary cardiomyopathy [12]. Cardiac arrhythmias get precipitated by alcohol consumption, be it acute or chronic. Heavy alcohol drinking is shown to impact the cardiovascular system in many ways, one of the most important among them being rebound hypertension [13]. Apart from congenital disorders of the cardiovascular system, it indeed is a very well-evident fact, which could be understood from the history of most of the patients diagnosed with cardiovascular disorders, that they used to consume a lot of alcohol for many years.

Impact of alcohol on the digestive system

Chronic alcoholism is found to have a very strong relationship with both acute pancreatitis and chronic pancreatitis. Chronic alcohol intake impairs the repair ability of the structures of the exocrine pancreas, thereby leading to pancreatic dysfunctioning [14]. Most of the patients diagnosed with pancreatitis have a strong history of chronic intake of alcohol. Liver diseases related to alcohol intake are known to humankind from the very beginning and probably are one of the oldest known forms of injury to the liver [15]. In liver diseases linked with alcohol, liver cirrhosis is a major concern. Statistics show that liver cirrhosis is one of the top 10 causes of death worldwide and this in itself indicates the severity of the same [16]. The changing lifestyle and also many people turning to prolonged alcohol intake for many years are contributing to the increased number of liver cirrhosis patients in the modern world. In liver cirrhosis patients, there occurs an increased severity of fibrosis due to the loss of parenchyma and fibrous scar proliferation [17]. Alcoholic liver disease (ALD) is an umbrella term which incorporates a wide range of injuries of the liver, spanning from simple steatosis to cirrhosis, and this also includes alcohol-related fatty liver disease (AFLD) and also alcoholic hepatitis [18]. Advancements in the diagnostic modalities have helped to diagnose ALD at an early phase and there is no doubt that newer and better investigations that have helped to detect more cases have led to a surge in the number of ALD patients on whole. Alcohol intake has a prominently bigger impact on the mortality of liver cirrhosis when compared with the morbidity [19]. A systemic review and meta-analysis suggests that women might be at a higher risk as far as developing liver cirrhosis is concerned even with little consumption of alcohol, as compared to men [20].

Impact of alcohol on the causation of cancer

Alcohol has much to do with cancers too and continuous research studies are conducted in order to find out the relationship between the two in detail. In a meta-analysis, it was found that women consuming alcohol had a later menopause onset, which is found to be associated with reduced cardiovascular disease risk and also all-cause mortality, but unfortunately, the happiness of this advantage gets compromised by the ironic fact that it has an increased risk of cancer (including ovarian and breast cancers) [21,22]. Large cohort studies, many meta-analyses, experimental research studies, etc are suggestive of the fact that the chronic intake of alcohol clearly increases colon and gastric cancer risk [23]. A causal association is also found between alcohol intake and cancers of the rectum, colon, liver, oesophagus, larynx, pharynx and oral cavity [24]. There are various theories put forward so as to understand the role of the consumption of alcohol in the development of cancer; there is suspicion that the rise in the number of alcohol users worldwide may be one of the reasons why the number of cancer patients is increasing at a global level. Chronic intake of alcohol may promote the genesis of cancer in many ways, some of the most notable ones being acetaldehyde (weak mutagen and carcinogen) production, cytochrome P450 2E1 induction associated oxidative stress, S-adenosylmethionine depletion/ which leads to global DNA hypomethylation induction, iron induction associated oxidative stress, retinoic acid metabolism impairment, etc [25].

Impact of alcohol on other systems

Apart from the systemic manifestations which do affect a particular system of the body, there are various disorders in which alcohol indirectly provides its crucial contribution. It is a common finding that one could perceive that alcohol is most of the time in the list of risk factors for various diseases. Alcohol has been found to adversely affect our immune system and the matter of concern as far as this issue is concerned is that immune responses are influenced by even moderate amounts of alcohol intake [26]. Alcohol affects innate immunity and also interferes with almost all the various aspects of the adaptive immune response. Alcohol is a key player in impairing anti-inflammatory cytokines and also promotes proinflammatory immune responses. The gastrointestinal biome is severely manipulated by the use of alcohol over a long period of time, which in turn is found to have a link with the establishment of various complications [27]. Alcohol and its metabolites are found to promote inflammation in the intestines and they do so through varied pathways [28]. Alcohol being a teratogen is documented to cause abnormalities of the brain, limbs, etc [29]. Multiple studies have been conducted across the globe to understand the effect of alcohol on humans; implications from certain such studies are put forth in Table 1. 

**Table 1:** Implications from various studies (sytemic review, meta-analysis, cohort study, prospective study, etc) regarding effects of alcohol consumption on the human body

Serial number	Objectives of the study	Type of study	Implications	Author, Country ( if applicable), Year [citations]
(1)	To provide a quantitative assessment of the association between the risk of cirrhosis of the liver and alcohol intake.	Systemic review and meta-analysis	Consumption of alcohol has a significantly large impact on the mortality of cirrhosis of the liver when compared with morbidity.	Rehm et al., July 2010 [19].
(2)	To systematically sum up the risk relationship between the incidence of liver cirrhosis and different alcohol consumption levels.	Systemic review and meta-analysis	Consuming about one drink per day in comparison to the long-term abstainers showed an increased risk in women for liver cirrhosis, but not in men.	Roerecke et al., October 2019 [20].
(3)	To analyse the impact of various drinking patterns in the causation of alcoholic cirrhosis of the liver.	A prospective cohort study	Recent alcohol consumption rather than earlier in life was found to be associated with a risk of alcoholic cirrhosis.	Askgaard et al., Denmark, May 2015 [30].
(4)	To investigate the relationship between the risk of colon and rectal cancer and the amount and type of alcohol.	Cohort study	Alcoholic intake significantly increases the risk of rectal cancer but this risk seems to be reduced with the inclusion of wine.	Pedersen et al., Denmark, June 2003 [31].
(5)	To examine the relationship between cancers of the upper digestive tract (oesophageal and oropharyngeal) and different types of alcoholic drinks.	Cohort study	A moderate wine intake does not probably increase the risk of cancer of the upper digestive tract, whereas a moderate intake of spirits or beers considerably increases the risk.	Grønbaek et al., Denmark, September 1998 [32].
(6)	To examine the relationship between death from all causes, coronary heart disease, and cancer and intake of different alcohol types.	Cohort study	Heavy drinkers who avoid wine are found to be at more high risk for death from all causes than heavy drinkers who do include wine in their alcohol intake.	Grønbaek et al., Denmark, September 2000 [33].
(7)	To investigate the long-term effects of the consumption of alcohol on morbidity and mortality.	Cohort study	The morbidity and mortality which is associated with different consumption levels of alcohol are associated with the same diseases, which suggests that alcohol might be one of the causative factors for these diseases.	Theobald et al., Sweden, November 2001 [34].
(8)	To provide updated quantification of association between the intake of alcohol intake and colorectal cancer.	Meta-analysis of the published observational studies	Showed evidence which strongly depicted an association between colorectal cancer risk and intake of alcohol.	Wang et al., May 2015 [35].
(9)	To investigate the association between atrial fibrillation risk in a prospective study of Swedish men and women and alcohol consumption.	Prospective study and a dose-response meta-analysis	Consumption of alcohol, even though at a moderate level, is one of the risk factors for atrial fibrillation.	Larsson et al., Sweden, July 2014 [36].
(10)	To examine the dose-response relationship between AF and consumption of alcohol.	Systemic review and meta-analysis	There is a causal impact of the consumption of alcohol on the onset of atrial fibrillation with a monotonic dose-response relationship.	Samokhvalov et al., December 2010 [37].
(11)	To investigate the effect of the consumption of alcohol on new-onset atrial fibrillation development in healthy individuals who are asymptomatic.	Multicenter study	In a healthy asymptomatic population, alcoholic drinking does increase the risk of new-onset atrial fibrillation in a dose-dependent manner which is regardless of the individual’s sex.	Cha et al., December 2020 [38].
(12)	To prospectively assess the association between incident atrial fibrillation (AF) among women and regular alcohol consumption.	Randomized control trial	Heavy consumption of two or more drinks per day was found to be associated with a small but statistically significant increased atrial fibrillation (AF) risk.	Conen et al., U.S., December 2008 [39].
(13)	To characterize the association between incident atrial fibrillation and chronic intake of alcohol.	Systemic review and meta-analysis	High intake of alcohol is associated with heightened atrial fibrillation (AF) risk across both genders.	Gallagher et al., November 2017 [40].

## Conclusions

Alcohol seldom leaves any system untouched as far as leaving its impression is concerned, spanning from single tissue involvement to complex organ system manifestations. Almost all the major organs that make up a human’s physiological being are dramatically affected by the overconsumption of alcohol. There is an enormous overall economic cost that is paid for alcohol abuse all over the world.
